# Endoscopic closure of refractory perforation after duodenal neuroendocrine tumor resection with multiple strategies

**DOI:** 10.1055/a-2960-2221

**Published:** 2026-10-26

**Authors:** Fang Xu, Zhao Yan, Chun Li, Rongxue Li, Zhe Jia, Jie Xing, Peng Li

**Affiliations:** 1 Department of GastroenterologyBeijing Friendship Hospital, Capital Medical UniversityBeijingBeijingChina; 2 State Key Laboratory of Digestive HealthBeijingChina; 3 National Clinical Research Center for Digestive DiseaseBeijingChina; 4 Beijing Key Laboratory of Early Gastrointestinal Cancer Medicine and Medical DevicesBeijingChina; 5 Department of General SurgeryBeijing Friendship Hospital, Capital Medical UniversityBeijingBeijingChina

A 43-year-old man was diagnosed with two neuroendocrine tumors (NETs)—one in the descending duodenum and another within the swollen, elevated duodenal ampulla—and both were successfully resected endoscopically despite the challenge. However, the duodenal ampulla is the common channel for the bile duct and pancreatic duct. The continuous chemical injury from the bile and pancreatic fluid was associated with impaired wound healing, contributing to a 4-cm perforation at the descending duodenal resection site, which was confirmed and repaired via emergency exploratory laparotomy. Unfortunately, due to tissue fragility, the perforation subsequently led to intra‑abdominal abscess with extension to the inguinal region.

To avoid second laparotomy, endoscopic perforation closure was performed using a dual action tissue clip (DAT) and an over-the-scope clip (OTS clip) to achieve reliable compression. A fully covered self‑expandable metal stent (FCSEMS) was also deployed near the duodenal papilla with small fenestrations created in the stent using an injection needle to protect the edematous inflamed tissue at the perforation margin from further chemical damage ( Fig. 1a–c , Video 1 ). In addition, early and adequate intra-abdominal infection drainage and therapeutic peritoneal lavage were applied, with appropriate antimicrobial therapy supported by a multidisciplinary team (MDT) and jejunal feeding tube insertion to maintain adequate nutrition and facilitate the restoration of bowel function. Ten weeks later, a small fistula adjacent to the OTS clip was observed. Thus, endoscopic debridement was performed by utilizing the DAT, OTS clip, and fenestrated FCSEMS again. At the three‑month follow‑up gastroscopy, fistula closure was confirmed, with no evidence of contrast extravasation.

**Fig. 1:**
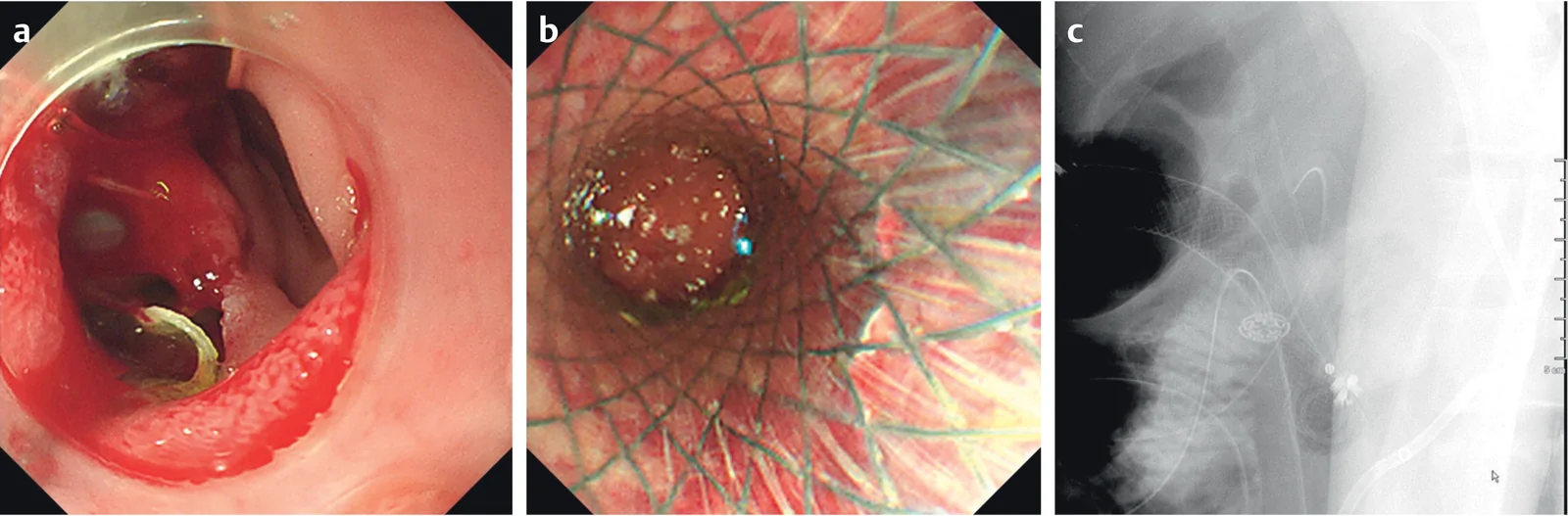
Endoscopic closure of duodenal perforation. ( **a** ) Gastroscopy revealed a perforation on the lateral side of the duodenal papilla. ( **b** ) The effect of the fenestrated FCSEMS. ( **c** ) A fluoroscopic view of the OTS clip and the FCSEMS. FCSEMS, fully covered self‑expandable metal stent; OTS clip, over-the-scope clip.

**Video 1** A triple-device strategy using the OTS clip, DAT and fenestrated FCSEMS for closure of perforation. DAT, dual action tissue clip; FCSEMS, fully covered self‑expandable metal stent; OTS clip, over-the-scope clip.


Bile and pancreatic secretions may impair tissue healing and contribute to refractory postoperative perforations in duodenal NET resection. 1​ ​2​ A triple-device strategy (the OTS clip to close the perforation, the DAT for tissue grasping, and the FCSEMS for pancreaticobiliary drainage) with MDT management enables complete closure after surgical failure without a second laparotomy.

## Declaration of GenAI Use

The authors confirm that no generative artificial intelligence was used in the preparation of this manuscript.

Endoscopy_UCTN_Code_CPL_1AH_2AG

## Data Availability

No data available.
